# Investigation of IL-17A Serum Levels in Patients with Nonmelanoma Skin Cancer

**DOI:** 10.1155/2021/5540163

**Published:** 2021-06-18

**Authors:** Mehdi Ghahartars, Fatemeh Sedaghat, Elham Khajavi, Amir Ali Nejat, Mahyar Malekzadeh, Abbas Ghaderi, Mohammad Javad Fattahi

**Affiliations:** ^1^Department of Dermatology, School of Medicine, Shiraz University of Medical Sciences, Shiraz, Iran; ^2^Shiraz Institute for Cancer Research, School of Medicine, Shiraz University of Medical Sciences, Shiraz, Iran

## Abstract

**Background:**

Role of interleukin 17A (IL-17A) in carcinogenesis and cancer growth is controversial. Although some researches support its antitumor activity, some others suggest that it promotes the growth and development of different types of cancer including skin cancer by activation of STAT3. Although the function of the cytokines such as IL-17A has been extensively studied in various types of cancer, nonmelanoma skin cancer (NMSC) has not received much attention. Therefore, the present study was aimed to investigate the serum levels of IL-17A in NMSC patients.

**Methods:**

This cross-sectional study was performed on 60 patients with basal cell carcinoma (BCC) and squamous cell carcinoma (SCC) as well as 57 age-sex matched healthy individuals as control group. Measurement of IL-17A serum levels in both case and control groups was performed by a commercially reliable sandwich enzyme-linked immunosorbent assay (ELISA) kit.

**Results:**

In this study, we observed that IL-17A serum levels in NMSC patients were significantly higher than the control group (*P* < 0.001). Also, both BCC and SCC patients had higher levels of IL-17A in their sera in comparison to the controls (*P*=0.001 and *P* < 0.001, respectively). However, there was no significant difference between SCC and BCC patients regarding serum levels of IL-17A.

**Conclusion:**

According to our results, it can be concluded that IL-17A may play a role in inducing the growth and progression of NMSC and it can be used as a therapeutic target in these patients in future.

## 1. Introduction

Skin cancer is one of the substantial health problems, which is the most common and most preventable malignancy in the world and its incidence does not show any signs of plateauing [1]. Skin cancer is commonly categorized as malignant melanoma and nonmelanoma skin cancer (NMSC) which is diagnosed more commonly than all other malignancies. The incidence of NMSC has increased by 4% annually, and currently, 2-3 million NMSCs are diagnosed worldwide each year [2]. There are several risk factors for all skin cancer types such as environmental, genetic, immunologic, and phenotypic factors [3, 4]. NMSC has two major histological subtypes, basal cell carcinoma (BCC) and squamous cell carcinoma (SCC). Approximately 80% of NMSC is BCC that proliferates slowly and most of the time does not metastasize, but it can destroy local underlying tissues while SCC is more destructive, invasive, and metastatic [5, 6]. NMSC is generally easily curable and rarely fatal. Despite such a good prognosis, there is evidence that other adverse health effects may be identified by NMSC, for instance, an association between NMSC and increased risk of other malignancies has been discovered [7, 8].

The interleukin 17A (IL-17A), previously known as cytotoxic T lymphocyte-associated antigen 8 (CTLA-8), is a T cell-derived proinflammatory cytokine which was introduced by Rouvier et al. in 1993 [9]. This cytokine is produced by T helper 17 (Th17) cells in response to their stimulation with IL-23. It is now known that various innate cells, including macrophages, dendritic cells (DC), *γδT* cells, and natural killer cells can secrete IL-17A [10]. IL-17A is a 155-amino acid, homodimeric, secreted glycoprotein with a molecular mass of 35 kDa. [11]. IL-17A expression tends to be upregulated during inflammation. IL-17A works alone or synergizes with other factors to activate multiple gene expression, including cytokines (IL-6, IL-19, IL-20, and IL-24), tumor necrosis factor-*α* (TNF-*α*), granulocyte colony-stimulating factor (G-CSF), chemokines, matrix metalloproteinase (MMP) 13, receptor activator of nuclear factor kappa-B ligand (RANKL), and antimicrobial peptides (lipocalin 2, b-defensin-2, S100A7, and S100A8/9) [12].

Although some researches support antitumor activity of IL-17A, some others suggest that it promotes the growth and development of various types of cancer including skin cancer [13]. Due to the controversial but remarkable role of IL-17A in cancer, in the present study, we investigated the serum levels of this cytokine in BCC and SCC patients to determine its role as a biomarker for skin cancer.

## 2. Material and Method

### 2.1. Study Population

This cross-sectional study was performed on 60 patients with BCC and SCC skin cancers. They were recruited from dermatology clinic of Faghihi Hospital affiliated with Shiraz University of Medical Sciences who had no previous history of treatment including surgery and anticancer drug or radiation therapy, as well as 57 age-sex matched healthy individuals as the control group without any history or evidence of cancer, autoimmune and genetic disorders in themselves, and their first-degree relatives and had no inflammatory or infectious diseases at least for the past 3 months. Approval for the study was obtained from the Ethical Committee of Shiraz University of Medical Sciences (IR.sums.med.rec.1397.163) and all patients and control group gave written informed consent before participation in the study. Demographic, medical history and clinical data were collected from medical documents or clinical examinations.

### 2.2. Enzyme-Linked Immunosorbent Assay (ELISA)

5 ml of the blood sample was obtained from all participants; the sera were separated and stored at −80°C until analysis. Measurement of IL-17A serum levels in both case and control groups was performed by a commercially reliable sandwich ELISA kit (MyBioSource, CA, USA) according to manufacturer's protocols and using the standard samples with known levels of IL-17A, provided by the manufacturer and presented as pg/ml.

### 2.3. Statistical Analysis

Statistical Package for Social Sciences (SPSS, version 22; Chicago, IL, USA) was used for data analysis. The normality of the information was analyzed using the Kolmogorov–Smirnoff test. Variables with normal distribution are presented as mean ± standard deviation (SD), or as median and first and third interquartile ranges (IQR). Frequencies are presented as percentages. Mann–Whitney U-test, Kruskal–Wallis test, and Dunn's post hoc test were used to analyze the differences among groups. To assess the value of IL-17A to discrimination of NMSC from the normal conditions, the area under the ROC curve (AUC) was determined and the best cut-off point for this marker as well as its sensitivity and specificity were calculated. In our study, *P* values of 0.05 or less were considered statistically significant.

## 3. Result

In the present study, a total number of 60 NMSC patients and 57 age-sex matched healthy individuals, as the control group, were investigated. The majority of the patients were male (*N* = 45, 75%) and the male-to-female ratio was 3 : 1 (45 : 15). The mean age of NMSC patients was 67.60 ± 12.82 years, and the most common diagnosis was SCC (*n* = 40, 66.67%). Most of the lesions were located in the sun-exposed area 54 (93.10%) and most of them were below 4 cm (66.7%). None of the patients had metastatic lesions.  Table 1 shows other clinicopathological characteristics of NMSC patients.

**Figure 1:**
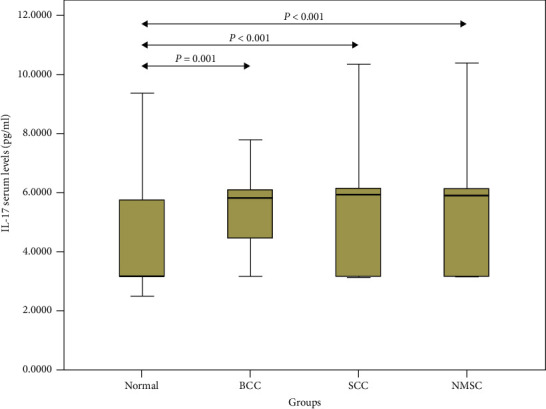
Box plot of IL-17 serum levels. The middle line represents the median. The ends of the boxes represent lower and upper quartiles and the ends of the whiskers represent the minimum and maximum. BCC, basal cell carcinoma; NMSC, nonmelanoma skin cancer; SCC, squamous cell carcinoma.

**Figure 2:**
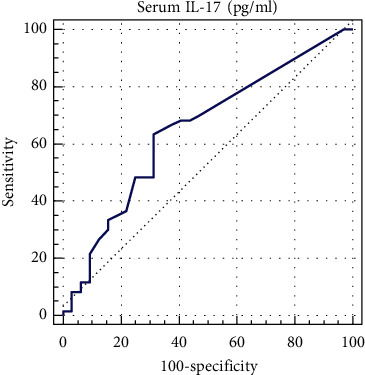
Area under the ROC curve analysis to determine the value of IL-17A in NMSC patients.

The median levels of IL-17A in the NMSC patients were 5.89 (3.20–6.13) pg/ml and in the control group was 3.20 (3.10–5.75) pg/ml respectively, which indicated significant differences (*P* < 0.001). In subgroup analysis according to pathologic diagnosis, serum levels of IL-17A were not different between SCC and BCC patients (*P*=0.793). However, patients with SCC had higher levels of IL-17A in their serum in comparison to the controls (5.95 (3.20–6.13) versus 3.20 (3.10–5.75) pg/ml; *P* < 0.001) (Figure 1). Investigation of the serum levels of IL-17A in BCC patients and the control group also demonstrated a statistically significant difference between these two groups (5.86 (3.81–6.12) versus 3.20 (3.10–5.75) pg/ml; *P*=0.001) (Figure 1). In this study, there was no significant relationship between serum levels of IL-17A and age (*P*=0.286) or sex (*P*=0.357) of the patients. There was no significant difference in serum levels of IL-17A in NMSC patients with sun-exposed lesions and patients with nonsun-exposed lesions (*P*=0.124). Furthermore, no relationship was found between number of lesions (single or multiple), tumor size, and IL-17A serum levels (*P*=0.667 and *P*=0.769, respectively) (Table 1). According to the ROC curve analysis (Figure 2), the measuring serum level of IL-17A could be a moderate indicator for distinguishing NMSC from normal conditions (AUC = 0.755; CI = 0.640–0.869). The best cut-off value of IL-17A for this differentiation was estimated to be 5.75 pg/ml, yielding a sensitivity of 63.3% and a specificity of 68.7%.

## 4. Discussion

Skin cancer is one of the most important cancers worldwide that imposes great economic and medical burdens. A conclusive link between inflammation and skin cancer has been demonstrated [14]. Inflammation is a mechanism of host defense and tissue regeneration which happens after tissue damage, infection, and stress. However, following persistent damage, chronic inflammation is capable of inducing DNA damage and genomic instability which causes the transformation of cancer-originating cells [15, 16]. Chronic inflammation contributes to approximately 20% of all human cancers [17]. Cytokines play an important role as mediators between tumor cells and the inflammatory microenvironment [18]. Although the role of the cytokines including IL-17A was extensively studied in different types of cancer, it has not received much attention in NMSC. Therefore, the present study investigated the serum levels of IL-17A in NMSC patients. We observed that IL-17A serum levels in NMSC patients were significantly higher than healthy individuals. Moreover, both BCC and SCC patients had higher levels of IL-17A in their serum compared to the controls. Our results are consistent with studies that indicate the elevated IL-17A expression in several human cancers, such as the colon, ovary, breast, prostate, and thyroid. [18–22]. Karabulut et al. investigated clinical outcomes of colorectal cancer (CRC) patients with serum IL-17 levels. They demonstrated that the baseline serum IL-17 levels were considerably higher in CRC patients. They also showed that the median serum IL-17 levels of patients with baseline poor grade tumors were significantly higher compared to good grade tumors, but there was no association between IL-17 and survival, so serum levels of IL-17 may be used as diagnostic markers in CRC patients [19]. Aotsuka et al. found that following changes occur in ovarian cancer patients, STAT3 is activated which produces IL-17A and TNF‐*α*, thereby eliciting an inflammatory response in patients. They suggest that increased Th17 counts and IL‐17 levels are potential biomarkers for poor prognosis in ovarian cancer [20]. In another study, Steiner et al. demonstrated that in the normal prostate, IL-17 expression was very low and restricted to lymphocytes, but in benign prostatic hyperplasia (BPH) and carcinoma specimens, IL-17 mRNA and protein expression were increased. They also showed that increased IL-17 expression under pathological conditions is accompanied by significant upregulation of some proinflammatory cytokines such as IL-6 and IL-8 [22].

In contrast with different kinds of malignancy, less examinations have been performed on serum levels of IL-17A in skin cancer, especially NMSC. Nardinocchi et al. have indicated that both SCC and BCC are penetrated with a high number of IL-17+ T lymphocytes. They also found that IL-17 can increase the proliferation and migration of the BCC and SCC cell lines and that IL-17 alone or in combination with TNF-*α* can induce the production of cytokines necessary for tumor progression [23]. As a matter of fact, IL-17 assists skin cancer development by activating STAT3 in tumor and stromal cells and promoting penetration of myeloid cells into the tumor microenvironment [23–25]. NF-*κ*B and STAT3 are fundamental for the development of inflammation-promoted cancer, such as skin cancer [26, 27]. In multiple mouse models, activation of NF-*κ*B in immune cells has been shown to result in the expression and production of multiple proinflammatory cytokines such as IL-17 which promotes the development of cancer [28, 29]. Wang et al. demonstrated that IL-17 disruption significantly reduced tumor genesis with decreased STAT3 activation in the tumor microenvironment which proves a critical role for the IL-17-STAT3 pathway in supporting cancer-associated inflammation in the tumor microenvironment [24, 30]. Ultra-violet radiation (UVR) seems to be a major contributor to skin cancer which promotes the transformation of skin cells by damaging cellular DNA. Furthermore, UVR acts as a link between skin cancer and inflammation, because its presence changes immune functions in the skin [31]. Exposure to UV light, for instance, leads to the upregulation of cyclooxygenase-2 (COX-2) in keratinocytes and increased production of prostaglandin E2 (PGE2), which causes inflammation of the skin tissue [32]. Studies have indicated that PGE2 in combination with IL-23 drives IL-17 production and Th17 cell differentiation and consequent pathogenesis in the skin [33].

The immune system functions contradictory, which can either facilitate or inhibit the growth of cancer. The definitive aim for cancer immunology research is to achieve a treatment outcome in which IL-17A and other cancer-promoting cytokine signals are blocked to reduce the growth of cancer cells and weaken resistance to therapy and their survival [34, 35]. IL-17A antibodies have already been found to be nontoxic and successful in treating various chronic inflammatory conditions such as rheumatoid arthritis, ankylosing spondylitis, inflammatory bowel disease (IBD), and psoriasis [36–39]. Further studies are needed to determine whether inhibiting proinflammatory cytokines can enhance the efficacy and/or safety of immune therapies for cancer.

## 5. Conclusion

According to our results, it can be concluded that IL-17A may play a substantial role in inducing the growth and progression of NMSC. Furthermore, IL-17A may be used as a therapeutic target in NMSC patients in the future, so to determine this effect, more studies are needed.

## Figures and Tables

**Table 1:** Clinicopathologic characteristics of NMSC patients and their respective IL-17A serum levels in each subgroup.

Variables		Number (valid percent)	IL-17A serum level (pg/ml)^1^	*P* value
Gender	Male	45 (75.0%)	5.64 (3.20–6.05)	0.357
	Female	15 (25.0%)	3.20 (3.00–6.02)	

Age	Over 50	55 (92.0%)	5.69 (3.20–6.05)	0.286
	Below 50	5 (8.0%)	3.20 (3.10–6.02)	

Type of NMSC	SCC^2^	40 (66.7%)	5.95 (3.20–6.013)	0.793
	BCC^3^	20 (33.3%)	5.86 (3.81–6.12)	

Tumor site	Sun-exposed	54 (93.10%)	5.82 (5.62–6.02)	0.124
	Nonsun-exposed	4 (6.90%)	5.44 (3.20–5.90)	

Lesions	Multiple	8 (13.3%)	5.85 (3.85–6.04)	0.667
	Single	52 (86.6%)	5.79 (3.20–6.16)	

Tumor size	≥4 cm	20 (33.3%)	6.02 (4.89–7.15)	0.769
	<4 cm	40 (66.7%)	5.89 (5.83–6.02)	

^1^Median (1^st^–3^rd^ interquartile); ^2^squamous cell carcinoma; ^3^basal cell carcinoma.

## Data Availability

The datasets generated and analyzed during the current study are not publicly available but are available from the corresponding author.
